# From awareness to action: do the food safety attitudes affect sustainable food consumption behaviors in university students?

**DOI:** 10.3389/fnut.2026.1750779

**Published:** 2026-03-16

**Authors:** Çağla Pınarlı Falakacılar, Gamzegül Bilginer Diler, Merve Terzi

**Affiliations:** 1 Department of Nutrition and Dietetics, Faculty of Health Sciences, Istanbul Gedik University, Istanbul, Türkiye; 2 Department of Nutrition and Dietetics, Institution of Graduate Studies, Istanbul Sağlık ve Teknoloji University, Istanbul, Türkiye; 3 Department of Nutrition and Dietetics, Faculty of Health Sciences, Istanbul Yeni Yuzyil University, Istanbul, Türkiye

**Keywords:** food safety, sustainability, sustainable food consumption, sustainable nutrition, university students

## Abstract

**Background:**

Ensuring food safety and promoting sustainable food consumption are increasingly important public health priorities, especially among young adults who are forming long-term dietary habits. This study explored how university students’ food safety attitudes relate to their sustainable food consumption behaviors.

**Methods:**

This cross-sectional study was conducted with 360 university students between May and September 2024. Validated scales were used to measure food safety attitudes (FSAS) and sustainable food consumption behaviors (SFCBS). Additional data collected included gender and body mass index (BMI). Statistical analyses evaluated differences between groups and correlations among key variables.

**Results:**

The findings showed that 83.3% of participants had adequate food safety attitudes. Female students scored significantly higher than males on both the food safety attitude and SFCBS scales, including subdimensions such as caring, assimilating, and shopping/cooking habits (*p* < 0.05). A moderate positive correlation was identified between FSAS and SFCBS, particularly for general nutritional behaviors (*r* = 0.446, *p* < 0.05). Additionally, positive correlations were found between SFCBS and FSAS subdimensions.

**Conclusion:**

Overall, the results indicate that female students exhibit stronger food safety attitudes and sustainable food consumption behaviors, and that fostering awareness of these practices during university years may contribute to healthier, more environmentally responsible lifestyles in line with global sustainability goals.

## 1 Introduction

Nutrition is the intake of nutrients that individuals need in sufficient quantities and at appropriate times to maintain and improve health and increase the quality of life. Adequate and balanced nutrition of individuals plays an important role in minimizing health problems related to nutrition, such as reducing the incidence of noncommunicable chronic diseases and preventing protein energy malnutrition, and vitamin-mineral deficiencies (1).

Recently, the effects of nutrition on human health and environment have become apparent (2). Many stages, including food production, food transportation and storage, cooking and waste management, contribute significantly to the formation of greenhouse gas emissions. Approximately 24%–30% of global greenhouse gas emissions originate from the agricultural sector; to achieve a significant reduction in greenhouse gas emissions, people need to change their diets and food choices and reduce food waste (3). Greenhouse gas emissions play an important role in global warming and, in this context, negatively affect planetary well-being (4). Food production is also one of the major reasons for water use. Ninety-two percent of all the water used is used for food production. Twenty-nine percent of the water used in agriculture is used directly or indirectly for animal production. Compared with plant products, animal products require more water per unit of energy (5).

Sustainable nutrition is highly important for food safety. Food safety is considered as the absence of physical, chemical and biological risk factors in a food (6). The phrases “ending hunger, achieving food security and good nutrition, supporting sustainable agriculture and ensuring a healthy and quality life at all ages” in the United Nations Sustainable Development Goals refer to food security. Sustainable nutrition is thought to play an important role in achieving sustainable development goals (7).

The lack of food safety and food security worldwide has led to malnutrition. Today, it is estimated that 3.1 billion people do not have access to healthy food and that approximately 670 million people will face hunger in 2030 (8). On the other hand, it is estimated that the world population will reach 10 billion by 2050. To meet the needs of this increasing population and achieve the Sustainable Development Goals, we need to use the resources we have in a sustainable way from today onward (9).

University years, a key phase of young adulthood, shape habits that impact future health and sustainability. Promoting sustainable dietary choices during this stage is vital for healthier lives and environmental protection (10). The lack of environmental awareness of the young population is noteworthy (11). University students represent a particularly suitable population group for examining the relationship between food safety attitudes and sustainable food consumption behaviors. During their university years, individuals typically take responsibility for their own food purchasing, preparation, and consumption decisions for the first time in their lives (12). Since nutritional habits and attitudes formed during young adulthood tend to continue into later life, this developmental stage is crucial (13). Therefore, understanding university students’ food safety attitudes and sustainable consumption behaviors is highly important for promoting long-term public health and environmental sustainability (14).

**Figure 1:**
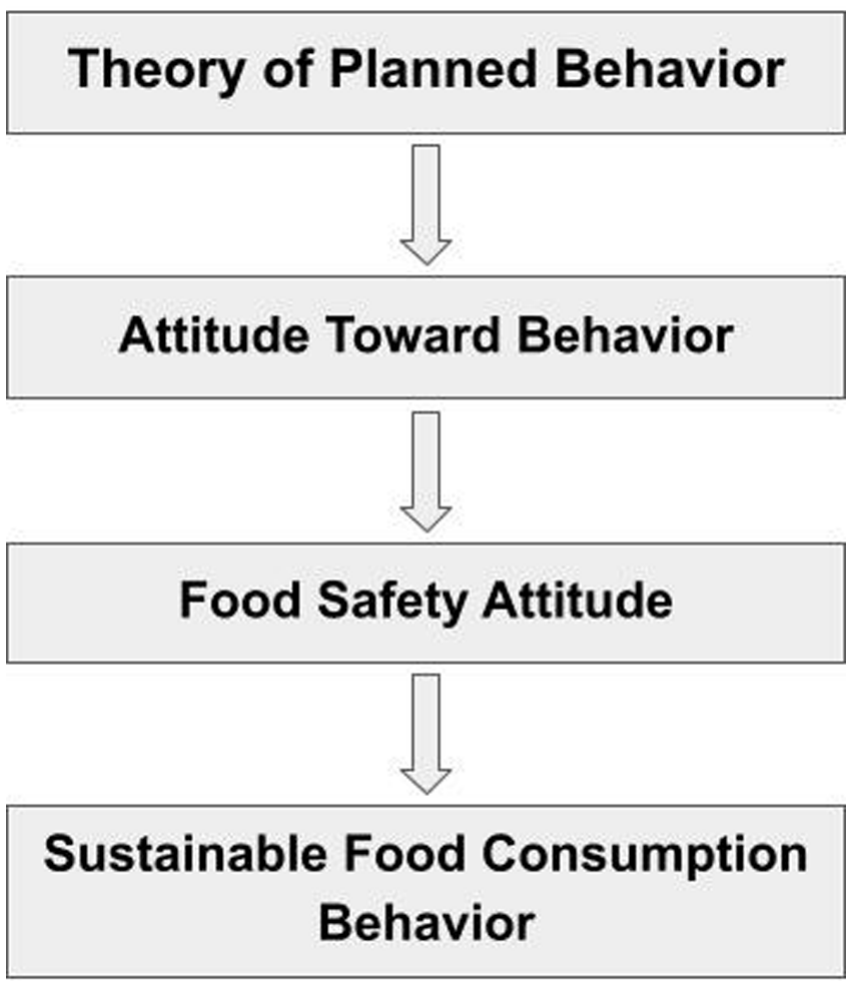
Conceptual framework based on the theory of planned behavior.

Theoretically, the relationship between food safety attitudes and sustainable food consumption behaviors can be explained within the framework of the Theory of Planned Behavior as shown in Figure 1. According to the Theory of Planned Behavior, individuals’ attitudes towards a behavior play a central role in shaping their behavioral intentions and, consequently, influencing their actual behavior (15). In this context, positive attitudes towards food safety, such as hygiene, safe food processing and trust in food systems, can increase individuals’ motivation to make more responsible and sustainable food choices (16, 17).

For these reasons, this study aims to determine the relationship between sustainable food consumption behaviors and food safety awareness levels among university students. The main hypothesis of this study is that more sustainable food consumption behaviors are associated with more positive food safety attitudes among university students. Investigating sustainable eating habits and food safety awareness among students will be beneficial both in determining the basic problems facing sustainable life and in raising awareness about sustainability and food safety. This study also aimed to raise awareness about the concepts of sustainable nutrition and food safety among university students.

There are limited number of studies examining the integrated relationship between food safety awareness and sustainable food consumption behavior within a single analytical framework, particularly among university students. While previous studies have addressed food safety awareness and sustainable consumption behaviors separately, evidence investigating their combined relationship within a specific population context remains insufficient. Therefore, this study aims to fill this gap by presenting context-specific evidence that contributes to a more comprehensive understanding of how food safety awareness is related to sustainability focused food choices among university students. In this respect, the findings of this study may contribute to the existing literature by offering empirical insights from a university student population.

## 2 Methods

### 2.1 Participants and study design

This cross-sectional study was conducted with volunteer participants studying at a private university between May and September 2024. The study was conducted with students from the Faculty of Health Sciences, Faculty of Sports Sciences, Faculty of Engineering, Faculty of Architecture and Design, Faculty of Economics, Administrative and Social Sciences. The study population consisted of undergraduate students enrolled in the selected faculties, with a total of 5.890 active students.

Where n represents the required sample size, N is the population size, p is the probability of occurrence (0.5), *q* is the probability of non-occurrence (0.5), t corresponds to the confidence level (1.96 for 95% confidence), and d represents the margin of error (0.05) (18).


$$n=\frac{\left[N\times t^{2}\times p\times q\right]}{\left[d^{2}(N-1)+t^{2}\times p\times q\right]}$$

The sample size was calculated to be a minimum of 357 people to represent the population with a 5% margin of error.

A non-probability quota sampling method was employed to reduce potential faculty-based sampling bias. To achieve a balanced representation, the target was to recruit approximately 60 students from each faculty. Participants were selected randomly within these predefined quotas.

Initially, a total of 386 students participated in the study. The study aimed to include individuals between the ages of 18 and 45 studying at the specified faculties. A total of 26 individuals who did not meet this requirement were excluded. Following data screening, 26 questionnaires were excluded due to incomplete responses or failure to meet the predefined age criteria. Consequently, the final analytical sample comprised 360 university students.

Before starting the study, ethical approval was obtained from the Istanbul Gedik University Non interventional Clinical Research Ethics Committee (Decision No: 2024/5-Barcode No: 334544, Date: 27.05.2024). This study was conducted in accordance with the principles of the Declaration of Helsinki. Google Forms questionnaires were sent to students who agreed to participate in the study. Each participant read and approved the informed consent form at the beginning of the study.

### 2.2 Data collection methods

The survey form sent via Google Forms contains a total of 3 sections. These sections include general information, the Food Safety Attitude Scale (FSAS), and The Sustainable Food Consumption Behavior Scale (SFCBS).

The general information section of the survey form includes questions about individuals’ general information such as age, sex, body weight, and height. The body mass index (BMI) value was obtained by dividing body weight (kg) by the square of height (m). Individuals’ BMI values were evaluated according to the WHO classification. A BMI less than 18.5 was considered underweight, a BMI between 18.5–24.9 was considered normal, a BMI between 25–29.9 was considered overweight, and a BMI of 30 and above was considered obese (19).

#### 2.2.1 The food safety attitude scale

The FSAS was developed by Memiş to assess individuals’ attitudes toward food safety (20). A Principal Component Analysis (PCA) was conducted on data obtained from the preliminary 30-item version of the scale to determine whether the instrument possessed a single- or multi-factor structure. Without constraining the number of factors, the analysis yielded a Kaiser–Meyer–Olkin (KMO) value of 0.87, indicating that the sample was highly adequate for factor analysis. Items with eigenvalues greater than 1.00 were retained in the scale.

To identify the factor structure, the varimax orthogonal rotation method was applied, and a factor-loading cut-off of 0.45 was adopted. In original scale, 12 items with weak performance were removed, and the remaining 18 items were grouped under two factors, namely: caring and assimilating.

The item total correlations ranged from 0.46 to 0.61 for the first factor and from 0.37 to 0.54 for the second factor, demonstrating that each item adequately discriminated participants’ attitudes toward food hygiene. The proportion of variance explained by the two factors was 23.5 and 18.5%, respectively, accounting for a total explained variance of 42%.

Reliability analyses showed strong internal consistency: the Cronbach’s *α* coefficients were 0.83 for the Caring sub dimension, 0.78 for the Assimilating sub dimension, and 0.85 for the total scale. These values indicate that the FSAS possesses a high level of internal reliability.

Developed by Memiş (2009), this scale has been validated as a reliable and valid instrument for measuring food safety attitudes among Turkish samples. Each item assesses participants’ sensitivity to food safety, adherence to hygiene rules, and safe eating habits (19).

In the present study, the original version of the FSAS was used to evaluate university students’ attitudes toward food safety. The scale consists of 18 items rated on a three-point Likert-type scale (1 = Disagree, 2 = Neutral, 3 = Agree). Higher total scores indicate more positive attitudes toward food safety. All FSAS items were retained and included in the analysis; no items were excluded. For the FSAS, attitude levels were interpreted according to Memiş (2009) as follows: ≤14 = negative attitude, 15–22 = partially positive attitude, and ≥23 = positive attitude.

#### 2.2.2 The sustainable food consumption behavior scale

The SFCBS was developed by Geiger et al. (21) to measure individuals’ environmentally and health-oriented food choices, and its Turkish validity and reliability study was conducted by Özen (2022) (22).

The Turkish adaptation, entitled “Sürdürülebilir Besin Tüketim Davranışı Ölçeği (SBTDÖ),” was validated in a sample of 272 adults aged 19–64 years (65.1% female, 34.9% male). According to the validation results reported by Özen (2022), the scale demonstrated a two-factor structure consisting of “purchasing preferences” and “nutritional preferences,” encompassing a total of 11 items. The confirmatory factor analysis (CFA) revealed satisfactory fit indices (*χ*^2^/df = 3.75, RMSEA = 0.10, CFI = 0.96, TLI = 0.95), confirming the construct validity of the two-dimensional model for Turkish samples (21).

Reliability analyses showed that the internal consistency coefficients were acceptable to high:

- Purchasing preferences subdimension: Cronbach’s *α* = 0.69.
- Nutritional preferences subdimension: Cronbach’s *α* = 0.81.

In addition, the test–retest reliability indicated high temporal stability (ICC = 0.88 for purchasing preferences, ICC = 0.86 for nutritional preferences, and ICC = 0.89 for the total scale).

In the present study, this validated Turkish version of the SFCBS was employed to evaluate university students’ sustainable food consumption behaviors. The scale items were rated on a five-point Likert-type scale ranging from 1 (strongly disagree) to 5 (strongly agree), with higher scores reflecting a stronger tendency toward sustainable food purchasing and nutritional behaviors. The total score was calculated as the mean of all items, and higher mean scores indicate more sustainable consumption behavior. All SFCBS items were retained and included in the analysis; no items were excluded. No cut-off points were defined in the original development or Turkish validation studies of the SCH, GNB, and SFCBS; therefore, no categorical classification was applied for these scales in the present study.

The FSAS and SFCBS were administered sequentially within the same online survey. All participants completed the scales in the same fixed order, with the FSAS administered first, followed by the SFCBS. As the two scales assess conceptually distinct constructs and were completed in a single session without any intervention, the sequential administration was not expected to influence participants’ responses.

### 2.3 Statistical analysis

All statistical analyses were performed using IBM SPSS Statistics version 25.0 (IBM Corp., Armonk, NY, United States). Prior to analysis, the dataset was examined for missing or inconsistent responses. Questionnaires containing incomplete or invalid data were excluded from the analyses. Since the rate of missing data was less than 5%, only valid and fully completed questionnaires were included in the evaluation. Missing data were handled using the list wise deletion method to ensure the accuracy of the statistical analyses.

Descriptive statistics were presented as frequency (*n*), percentage (%), mean (M), and standard deviation (SD). The normality of quantitative variables was tested using the Kolmogorov–Smirnov test. As the data were normally distributed (*p* > 0.05), parametric statistical methods were employed in the study.

Differences between two independent groups (female and male students) were analyzed using the independent samples *t*-test. Comparisons of mean scores across the three Body Mass Index (BMI) categories (underweight, normal, overweight) were performed using one-way analysis of variance (ANOVA).

Relationships between continuous variables were examined using the Pearson correlation analysis. The strength of the correlation coefficients (*r*) was interpreted according to the following criteria (16); *r* ≤ 0.30: low-level correlation, 0.30 < *r* < 0.70: moderate-level correlation, *r* ≥ 0.70: high-level correlation.

All statistical tests were evaluated at a 95% confidence level, and a *p*-value of < 0.05 was considered statistically significant (18).

## 3 Results

In this study, conducted with a total of 360 students from different faculties, the distribution of the demographic characteristics and food safety attitudes scores of university students are presented in Table 1. Among the participants, 280 (77.8%) were female, and 80 (22.2%) were male. When the distribution of participants according to their food safety attitudes was examined, the vast majority (83.3%) had an adequate attitude, and 16.7% had a partially adequate attitude. The scores of the students from the Food Safety Attitude Scale and the Sustainable Food Consumption Behavior Scale are given in Table 2. When evaluated according to gender, female students had significantly higher scores on the food safety attitudes scale total score and its sub dimensions (caring and assimilating) than male students (*p* < 0.05). Compared with male students, female students had significantly higher scores on the SFCBS and its sub dimensions (shopping and cooking habits and general nutritional behavior) (*p* < 0.05) (Table 3).

**Table 1:** Distribution of demographic characteristics of participants.

Variables		Number of participants	Percentage
Gender	Female	280	77.8
	Male	80	22.2
BMI	Underweight	43	11.9
	Normal	235	65.3
	Overweight	82	22.8

Variables	Number of participants	Mean ± SD	Min-Max
Age	360	23.81 ± 6.46	18–45
Body weight (kg)	360	63.11 ± 12.59	43–82
Height (cm)	360	167.31 ± 7.97	150–193
BMI	360	22.45 ± 3.56	14.88–29.72

Min-Max, Minimum-Maximum; SD, Standard deviation.

**Table 2:** Evaluation of participants’ scores from the scales.

Variables	Mean ± SD	Min-Max
Caring	25.21 ± 2.72	15–27
Assimilating	21.41 ± 4.50	9–27
FSAS score	23.31 ± 3.04	15.5–27
SCH	2.93 ± 0.71	1–5.11
GNB	2.96 ± 0.41	1.88–4.25
SFCBS score	2.95 ± 0.45	1.63–4.04

FSAS, Food safety attitude scale; SCH, Shopping and cooking habits; GNB, General nutrition behavior; SFCBS, Sustainable food consumption behavior scale; SD, Standard deviation; Min-Max, Minimum-maximum.

**Table 3:** Comparison of scale scores by gender groups (independent samples *t*-test results).

Variables	Gender	*N*	Mean ± SD	*t*	df	*p* value
Caring	Female	280	25.64 ± 2.26	5.891	358	<0.001
	Male	80	23.70 ± 3.55			
Assimilating	Female	280	22.39 ± 4.00	8.526	358	<0.001
	Male	80	17.95 ± 4.48			
FSAS score	Female	280	24.02 ± 2.64	9.191	358	<0.001
	Male	80	20.83 ± 3.08			
SCH	Female	280	2.99 ± 0.71	2.853	358	0.005
	Male	80	2.73 ± 0.68			
GNB	Female	280	3.04 ± 0.41	7.539	358	<0.001
	Male	80	2.68 ± 0.22			
SFCBS score	Female	280	3.02 ± 0.45	5.589	358	<0.001
	Male	80	2.71 ± 0.38			

Independent samples *t*-test was used to compare the mean scores between female and male students. Values are presented as Mean ± SD. *p* < 0.05 indicates statistical significance. FSAS, Food safety attitude scale; SCH, Shopping and cooking habits; GNB, General nutritional behaviors; SFCBS, Sustainable food consumption behavior scale.

Table 4 shows whether there was a relationship between the participants’ BMI values and the scores they received from the scales. No significant relationship was found between the classification of university students according to their BMI values and the total and sub scores of the food safety attitudes scale (*p* > 0.05). Again, no significant difference was found between the students’ sustainable food consumption behavior scale total score and sub scores and their BMI values (*p* > 0.05). An evaluation of the relationship between food safety attitudes scores and sustainable food consumption behaviors is given in Table 5. There is a moderate positive relationship between the FSAS and SFCBS and general nutrition behaviors (GNB) (*r* = 0.405, *r* = 0.446, *p* < 0.05, respectively). There is a weak positive relationship between the SFCBS and shopping and cooking habits (SCH) (*r* = 0.261, *p* < 0.05).

**Table 4:** One-way ANOVA results according to BMI groups.

Variables	BMI group	*N*	Mean ± SD	*F* (df₁, df₂)	*p* value
Caring	Underweight	43	25.02 ± 2.65	1.358 (2, 357)	0.258
	Normal	235	25.38 ± 2.81		
	Overweight	82	24.83 ± 2.49		
Assimilating	Underweight	43	20.70 ± 5.15	0.948 (2, 357)	0.389
	Normal	235	21.63 ± 4.21		
	Overweight	82	21.15 ± 4.94		
FSAS score	Underweight	43	22.86 ± 3.49	1.401 (2, 357)	0.248
	Normal	235	23.50 ± 2.92		
	Overweight	82	22.99 ± 3.13		
SCH	Underweight	43	2.97 ± 0.64	0.139 (2, 357)	0.870
	Normal	235	2.93 ± 0.73		
	Overweight	82	2.90 ± 0.68		
GNB	Underweight	43	3.01 ± 0.44	0.863 (2, 357)	0.423
	Normal	235	2.94 ± 0.41		
	Overweight	82	3.00 ± 0.39		
SFCBS score	Underweight	43	2.99 ± 0.46	0.294 (2, 357)	0.746
	Normal	235	2.94 ± 0.44		
	Overweight	82	2.95 ± 0.48		

One-way analysis of variance (ANOVA) was conducted to compare the mean scores across BMI categories (underweight, normal, and overweight).

No statistically significant differences were found between groups for any of the variables (*p* > 0.05). *Post hoc* comparisons were performed using the least significant difference (LSD) test.

FSAS, Food safety attitude scale; SCH, Shopping and cooking habits; GNB, General nutritional behaviors; SFCBS, Sustainable food consumption behavior scale.

**Table 5:** Correlation between FSAS and SFCBS scores.

Variables		FSAS	Caring	Assimilating	SFCBS	SCH	GNB
FSAS	*r*	1					
	*p*						
Caring	*r*	0.730*	1				
	*p*	0.000					
Assimilating	*r*	0.911*	0.382*	1			
	*p*	0.000	0.000				
SFCBS	*r*	0.405*	0.268*	0.385*	1		
	*p*	0.000	0.000	0.000			
SCH	*r*	0.261*	0.229*	0.215*	0.901*	1	
	*p*	0.000	0.000	0.000	0.000		
GNB	*r*	0.446*	0.199*	0.483**	0.658*	0.265*	1
	*p*	0.000	0.000	0.000	0.000	0.000	

FSAS, Food safety attitude scale; SCH, Shopping and cooking habits; GNB, General nutrition behavior; SFCBS, Sustainable food consumption behavior scale; *r*, Pearson correlation coefficient; *p*, *p* value; *, *p* < 0.05.

There is a moderate level positive relationship between the SFCBS and assimilation (*r* = 0.385, *p* < 0.05), and a weak positive relationship between the SFCBS and caring (*r* = 0.268, *p* < 0.05). There is a weak positive relationship between caring and SCH and GNB (*r* = 0.229, *r* = 0.199, *p* < 0.05, respectively). A weak positive relationship was found between assimilation and SCH (*r* = 0.215, *p* < 0.01), and a moderate-level positive relationship was found between assimilation and GNB (*r* = 0.483, *p* < 0.01).

A multiple linear regression analysis was conducted to determine whether FSAS independently predicts SFCBS when controlling for gender, faculty, and body mass index (BMI) (Table 6). The overall model was statistically significant, *F*(4,355) = 19.26, *p* < 0.001, explaining 17.8% of the variance in sustainable food consumption behavior scores (Adjusted *R*^2^ = 0.169). Food safety attitude scores were positively associated with sustainable food consumption behavior scores (*β* = 0.346, *p* < 0.001).

**Table 6:** Multiple linear regression analysis associating SFCBS.

Predictor	B	SE	*β*	*t*	*p* value
Constant	1.918	0.237	–	8.097	<0.001
FSAS	0.052	0.008	0.346	6.404	<0.001
Gender	−0.145	0.060	−0.133	−2.432	0.016
Faculty	0.002	0.010	0.010	0.201	0.841
BMI	−0.001	0.038	−0.002	−0.038	0.970

*F*(4,355) = 19.26, *p* < 0.001, Adjusted *R*^2^ = 0.169, Std. Error = 0.413. Dependent variable: SFCBS, sustainable food consumption behavior scale.

Gender was significantly associated with sustainable food consumption behavior scores (*β* = −0.133, *p* = 0.016). In contrast, faculty and BMI did not significantly predict sustainable consumption behavior (*p* > 0.05). These findings demonstrate that food safety attitude is an independent and robust determinant of sustainable food consumption behavior, regardless of gender, academic department, or body weight status.

## 4 Discussion

### 4.1 Overview of food safety and sustainable food consumption

Food safety and food security are key elements of healthy and sustainable food production. A healthy and sustainable food production system must include both food security and safety as essential elements to maintain long-term food availability and public health. While food security guarantees that people always have access to enough safe, nourishing food, food safety focuses on preventing contamination and foodborne illnesses through appropriate handling, storage, and processing (23). In this context, this study investigated the relationship between sustainable food consumption behavior and food safety awareness among university students from different faculties.

### 4.2 Sociodemographic and anthropometric factors in relation to SFCBS and FSAS

This research revealed that the sustainable food consumption behavior score was significantly higher for female students than for male students (*p* < 0.05). When compared with studies from different countries, our findings show both similarities and contextual differences. Research conducted in European and East Asian university populations has similarly reported higher food safety attitudes and more sustainable food consumption behaviors among female students. However, the magnitude of these gender differences appears to be smaller in countries with higher gender equality and more shared household food responsibilities. In contrast, studies from Middle Eastern and some developing countries tend to report larger gender gaps, likely reflecting traditional gender roles in food preparation and household food management. These cross-cultural differences suggest that social norms and cultural expectations play an important role in shaping both food safety attitudes and sustainable consumption behaviors (24–26). This may be related to the fact that in societies women having higher levels of nutritional knowledge and healthier food preferences, being more sensitive to their physical appearance, being more responsible for the nutrition of their family and children, spending more time in the kitchen, and being more interested in nutrition than men (27, 28). Previous research indicates that university students’ dietary habits are often influenced by factors such as living arrangements, academic workload, and accessibility to healthy food options (29, 30). Studies from various countries have shown similar trends, with students commonly exhibiting irregular meal patterns and low consumption of fruits and vegetables (31). Compared to these findings, the participants in the present study demonstrated relatively positive nutrition behaviors, which may reflect growing awareness of food safety and sustainability issues within the university context.

Sustainable eating behaviors have been shown to be associated with an increased level of food literacy and a lower BMI (32). It has been reported that an increase in sustainable eating behaviors is associated with a decrease in the risk of overweight and obesity (33). In this study, there was no relationship between BMI and sustainable eating behaviors (*p* > 0.05). Considering that the mentioned studies were conducted in adults, this result was likely obtained because university students are disadvantaged at this point. Unhealthy eating patterns, including frequent fast food consumption, and sedentary behaviors are common among university students (34). However, as variables such as fast food consumption frequency and sedentary behavior were not directly measured in the present study, no definitive conclusions can be drawn regarding their potential impact on BMI. Therefore, the absence of a significant association may indicate that BMI is not a direct indicator of sustainable food consumption behaviors in this population, or that additional unmeasured factors may play a role.

A study evaluating the Sustainable and Healthy Eating Behavior Scale scores of different generations revealed that individuals born between 1965 and 1979 scored 4.56 ± 0.78, those born between 1980 and 1999 scored 4.27 ± 0.83, and those born in 2000 and later scored 4.02 ± 0.93 (35). Younger individuals show less compliance with sustainable and healthy eating behaviors. Considering that the mean age of the university students, who constitute the population of this study, was 23.81 ± 6.46, it is a possible that lower scores were obtained than those of the study groups, including older age groups. This suggests that extra practices and education should be provided to increase sustainable nutrition behaviors among university students.

The positive associations between the caring and assimilating sub dimensions of the FSAS and general nutritional behaviors suggest that greater emphasis on food safety and the integration of safety knowledge into daily routines are linked to healthier, sustainability-oriented dietary practices. Together, these subdimensions may help explain the observed relationship between overall food safety attitudes and general nutrition behaviors (36).

Food safety is a critical aspect of public health, influencing individuals across all demographics, regardless of age, ethnicity, gender, or socioeconomic status. Nonetheless, gender often emerges as a significant factor in discussions surrounding food safety. Women who frequently serve as primary caregivers and food preparers within households, are commonly linked to responsibilities associated with ensuring food safety (37). This agreement is also observed in our study, which reveals that women have higher positive food safety attitudes scores than men. In addition, women have more positive behavior for both the caring and assimilating subdimensions of the FSAS, which indicates better food safety attitudes. According to a study done on Jordanian homes, women typically score higher than men on the knowledge, attitudes, and behaviors (KAP) scale related to food safety. Only 5.6% of the males had comparable KAP scores, whereas 18.0% of females showed better scores. This implies that women might be more conscious of and have more favorable opinions about food safety precautions (38). This is mostly because of handling preparation, cooking and serving. All these processes require women to be sure about the food safety because of the theory of planned behaviors. This theory explains why more favorable attitudes and behavioral controls are seen in women (39). Another possible explanation for women’s superior food safety awareness is that they are more likely to read and use food labels than males are. Food labels, which contain safety and nutritional data, are essential for making educated food decisions. A study revealed that women are more inclined to look at ingredient lists, nutritional information, and expiration dates, indicating greater care for the health and welfare of their families (40, 41).

### 4.3 A theoretical perspective on food safety attitudes and sustainable food consumption

Food safety attitudes and knowledge are linked to each other. A higher scores of food safety knowledge enhances individuals’ ability to make informed choices regarding food selection while shopping, including identifying fresher items, opting for products with longer expiration dates, avoiding bulk purchases when unnecessary, and understanding food labels (42). In our study, better food safety knowledge was significantly positively correlated with better shopping behavior (*p* < 0.05). In one systematic review, researchers revealed that food safety knowledge may influence consumer choices, resulting in a lower incidence of food-borne illnesses (43). In one cross-sectional study, the importance of food safety knowledge, attitudes and behaviors was emphasized. It has been reported that better food safety knowledge may have a direct and indirect effects on consumer choices, resulting in better food selection and health status (44). A positive correlation was observed between food safety knowledge and cooking behaviors in this study (*p* < 0.05). In a study assessing food safety knowledge in university students, increased cooking behaviors were associated with higher food safety knowledge scores (45). This result also highlights the result of our study.

Food waste reduction, improved and sustainable production, minimal chemical use, innovative packaging, and local and seasonal food systems are all aspects of sustainability and food safety that are mutually dependent (40, 41). Additionally, by reducing the possibility of diseases or toxins spreading throughout the food chain and lowering the usage of pesticides, antibiotics, and other substances of concern, food safety contributes to a sustainable food system (46–51). This study indicates that there is a positive correlation between food safety attitudes and improved sustainable food consumption behaviors (*r* = 0.405, *p* < 0.05). Furthermore, there was a favorable correlation between sustainability and the subdimensions of food safety attitudes toward caring and assimilating. These results are mostly related to the connection between food safety and sustainability with respect to mutual targets. The internal mechanisms linking food safety attitudes to sustainable food consumption can be explained through the subdimensions of caring and assimilating. The ‘caring’ dimension reflects heightened concern for food quality, hygiene, and health, which may lead individuals to prefer local and seasonal foods perceived as fresher and less processed, as well as more plant-based options associated with lower health and environmental risks. The ‘assimilating’ dimension reflects the integration of food safety knowledge into daily practices, such as improved meal planning, appropriate food storage, attention to expiration dates, and more mindful purchasing decisions, all of which contribute to reducing food waste. Together, these mechanisms help explain the observed positive association between food safety attitudes and sustainable food consumption behaviors.

Socioeconomic factors, particularly income, are known to influence both food safety practices and sustainable food choices. Studies have shown that individuals with higher income levels tend to demonstrate stronger food safety attitudes, possibly due to better access to information, education, and safe food options (52). Conversely, students from lower-income backgrounds may experience constraints that limit their ability to prioritize food safety and sustainability over economic concerns. Among individuals living in low and middle-income countries, a study shows that increased consumer concern about food safety increases the consumption of processed and packaged foods and decreases sustainable food consumption behaviors. Therefore, increased knowledge of food safety may also be associated with a decrease in sustainable food consumption behaviors. When comparing our findings with international literature, most studies conducted in high-income countries support a positive association between food safety awareness and sustainable food consumption behaviors, similar to our results. However, some studies from low- and middle-income countries have reported that increased food safety concerns may lead consumers to prefer more processed and packaged foods, which may reduce sustainability-oriented choices. These differences may be explained by variations in food availability, trust in local food systems, market conditions, and socioeconomic constraints (43, 53). Considering that private university students, who constitute the sample of this study, are more financially advantaged, income level may be important in the formation of a positive relationship between food safety knowledge scores and sustainable eating behaviors.

### 4.4 Strengths and limitations

This study has several strengths. First, to the best of our knowledge, this is the first study assessing the relationship between food safety awareness and sustainable food consumption behavior, especially in university students. This current study discusses food safety, sustainability, and consumption - three important topics in current studies and policy because of public health issues and climate change. Studying university students is essential since they will make decisions in the future and have the power to affect sustainability initiatives.

This study has several limitations that should be acknowledged. First, the data were collected from students enrolled at a single private university, which introduces potential selection bias and may limit the generalizability of the findings to students from public universities or different institutional contexts. In Turkey, students attending private universities typically come from relatively higher socioeconomic backgrounds, which may influence both food safety awareness—through better access to information—and sustainable consumption behaviors—through greater purchasing power. In addition, the relatively small sample size, the focus on a specific age group, and the restriction to a single city and institution further limit the external validity of the results. Furthermore, the gender distribution in the sample is unbalanced, which may also partially affect generalizability. From the outset, this study was designed as an exploratory and analytical investigation aimed at examining possible relationships rather than drawing conclusions at the population level. Given the cross-sectional design, these findings reflect associations rather than causal relationships. Longitudinal studies are needed to establish temporal precedence and causal direction (54).

Finally, data were collected using self-reported questionnaire methods, which may introduce subjectivity and potential response bias. Although validated measurement instruments were used, the reliance on self-reported data may affect the objectivity of the findings. Future studies are encouraged to adopt mixed method approaches by combining subjective measures with objective data sources, such as observational methods or behavioral assessments, to enhance the robustness and credibility of the conclusions. In addition, future research is recommended to include larger and more balanced samples covering different regions, universities, and age groups with diverse socioeconomic characteristics.

## 5 Conclusion

This study investigated the connection between university students’ awareness of food safety and sustainable food consumption practices, offering important new information on how these two factors interact when making decisions about food.

The key conclusions of the present study are summarized below:

- Food safety attitude is an independent and significant associated factor of sustainable food consumption behavior, even after controlling for gender, faculty, and BMI.
- Female students exhibited significantly higher food safety attitude scores and sustainable food consumption behavior scores compared with male students, suggesting gender-related differences in both awareness and behavior.
- A positive relationship was identified between food safety attitudes and sustainable food consumption behaviors. Higher food safety attitude scores were associated with higher levels of sustainable food purchasing, cooking habits, and general nutritional behaviors.

Furthermore, the findings of this study suggest that food safety awareness may be associated with sustainable food consumption behaviors among university students. From a practical perspective, universities may consider integrating short modules on food safety and sustainable consumption into orientation programs or general education courses. Campus food services may implement sustainability labeling systems to increase awareness at the point of purchase.

In addition to adding to the expanding corpus of research on sustainable consumption, this study lays the groundwork for future investigations into these relationships in a variety of settings. More comprehensive studies in different populations are needed.

## Data Availability

The raw data supporting the conclusions of this article will be made available by the authors, without undue reservation.
